# Identification and Validation of Four Novel Promoters for Gene Engineering with Broad Suitability across Species

**DOI:** 10.4014/jmb.2103.03049

**Published:** 2021-06-29

**Authors:** Cai-Yun Wang, Li-Cheng Liu, Ying-Cai Wu, Yi-Xuan Zhang

**Affiliations:** School of Life Science and Biopharmaceutics, Shenyang Pharmaceutical University, Shenyang 110016, P.R. China

**Keywords:** Promoter, cross-species bacteria, *gfp*, *Ketogulonicigenium*, *minCD*, sorbose dehydrogenase

## Abstract

The transcriptional capacities of target genes are strongly influenced by promoters, whereas few studies have focused on the development of robust, high-performance and cross-species promoters for wide application in different bacteria. In this work, four novel promoters (P_k.r_*tufB*, P_k.r_1, P_k.r_2, and P_k.r_3) were predicted from *Ketogulonicigenium robustum* and their inconsistency in the -10 and -35 region nucleotide sequences indicated they were different promoters. Their activities were evaluated by using green fluorescent protein (*gfp*) as a reporter in different species of bacteria, including *K. vulgare* SPU B805, *Pseudomonas putida* KT2440, *Paracoccus denitrificans* PD1222, *Bacillus licheniformis* and *Raoultella ornithinolytica*, due to their importance in metabolic engineering. Our results showed that the four promoters had different activities, with P_k.r_1 showing the strongest activity in almost all of the experimental bacteria. By comparison with the commonly used promoters of *E. coli* (*tufB*, lac, lacUV5), *K. vulgare* (P*sdh*, Psndh) and *P. putida* KT2440 (JE111411), the four promoters showed significant differences due to only 12.62% nucleotide similarities, and relatively higher ability in regulating target gene expression. Further validation experiments confirmed their ability in initiating the target *minCD* cassette because of the shape changes under the promoter regulation. The overexpression of sorbose dehydrogenase and *cyt*ochrome *c551* by P_k.r_1 and P_k.r_2 resulted in a 22.75% enhancement of 2-KGA yield, indicating their potential for practical application in metabolic engineering. This study demonstrates an example of applying bioinformatics to find new biological components for gene operation and provides four novel promoters with broad suitability, which enriches the usable range of promoters to realize accurate regulation in different genetic backgrounds.

## Introduction

Microorganisms have been widely used to produce various products such as amino acids [1, 2], biofuels [3] and pharmaceuticals [4] in the metabolic engineering practices, because the techniques are able to preserve some unique metabolic pathways to produce specific products, which further enhances their value by introducing new metabolic pathways and genetic control [5-7]. However, when a pathway or genetic part is transferred from one strain to another, failures usually take place unexpectedly, so many efforts are usually required to sustain function. The causes are mainly because the biological blocks are usually affected by host cells, and the altered activities usually result in the failure of the biological processes. For example, when the well-built T7 system was used in *Halomonas* sp. TD01, inexplicable failure occurred despite multiple troubleshooting attempts [8]. This phenomenon indicates that novel functional biological blocks are still highly in demand for genetic engineering. In synthetic biology, cells are often needed to express a certain protein at a specific intensity; however, the difficulty is in controlling the expression up to a target level regardless of genetic contexts. One of the widely used ways is to adjust the strength of the corresponding promoters. For example, the *tufB* promoter of *Escherichia coli* (*E. coli*_*tufB*) did not have enough activity to initiate the *ga2dh* gene expression for high-level production of 2-keto-D-gluconic acid in *Gluconobacter oxydans* DSM 2003, but replacement of *E. coli*_*tufB* by the intronic promoter gHp0169 of *G. oxydans* DSM 2003 resulted in a 2-fold increase of the yield [9]. Shen *et al*.[10] reported the usage of the optimized promoters to initiate *orfZ* gene expression to produce Poly(3-hydroxybutyrate-co-4-hydroxybutyrate) in *H. bluephagenesis*, where the best result reached 100 g/l cell dry weight with productivity of 1.59 g/l/h, which was 60% higher than that of the original strain. Hence, developing a series of specific promoters for gene engineering is a necessary prerequisite in fine-tuning gene expression. Moreover, the stability of a metabolic system is also important when engineering a novel pathway or altering an existing one to produce target products. An unstable system may result in the depletion of essential metabolites or the accumulation of toxic intermediates, leading to the loss of desired production or the death of engineered target cells [11]. Therefore, promoters with a broad range of transcriptional capacities are always necessary, depending on the purpose of the gene expression.

*Ketogulonicigenium robustum* SPU_B003 is a novel strain with independent growth characteristics and higher yield of 2-keto-L-gulonic acid (2-KGA) , the precursor of vitamin C [12], so it was a good chassis for genetic engineering in realizing one-step fermentation of 2-KGA. However, a problem in genetic operating with this strain is the lack of robust, high-performance promoters to implement biological process control. Therefore, new genetic building blocks with wide host applicability are much needed to fully realize the potential of *Ketogulonicigenium* and other microorganisms. In this study, four putative promoters (P_k.r_1, P_k.r_2, P_k.r_3, P_k.r_*tufB*) were isolated and characterized based on the transcriptome and genome analysis of *K. robustum* SPU_B003, and their availability and intensity were evaluated by the whole cell fluorescence intensity of green fluorescent protein (GFP). The subsequent cross-species evaluation was conducted in *K. vulgare* SPU B805, *Pseudomonas putida* KT2440, *Paracoccus denitrificans* PD1222, *Bacillus licheniformis* and *Raoultella ornithinolytica* due to their good qualities and immense importance in metabolic engineering. *K. vulgare* SPU B805, another 2-KGA-producing strain, can convert L-sorbose to 2-KGA with high efficiency when co-cultured with companion strain [13]. *P. putida* KT2440 is a good chassis for the production of various industrial products such as n-butanol [14], vanillin [15], and ethanol [16] because of its versatile metabolism and low nutritional requirements [17]. *P. denitrificans* PD1222, a soil-denitrifying bacterium, is considered as one of the best sources for polyhydroxyalkanoate (PHA) production because the strain can accumulate high yield in the cells [18], and can use a wide variety of industrial wastes as carbon sources to produce target products [19, 20]. *B. licheniformis*, as a generally regarded as a safe (GRAS) strain with the qualified characteristics of fast growth rate and strong sugar consumption, is usually used to produce 2,3-butanediol (BDO) [21], and is also a good chassis strain for the production of acetoin, a common additive in the food industry and a building block for chemical materials [22]. *R. ornithinolytica* can be used to produce 2,5-furandicarboxylic acid (FDCA), an important renewable biotechnological building block. Moreover, *R. ornithinolytica* has great biotechnology potential for its ability to produce biomolecules of industrial significance, such as 2,3-butanediol (2,3 BD) [23], pullulanase [24] and 2,5-furandicarboxylic acid (FDCA) [25]. These strains, as flexible cell factories, are of great potential in industrial biotechnology and have been selected for cross-species evaluation of the new promoters. In addition, the activity of the four identified promoters was further confirmed by the morphological changes through the target *minCD* cluster expression. The enhancement of 2-KGA yield by overexpression of sorbose dehydrogenase and *cyt*ochrome *c551* under promoters P_k.r_1 and P_k.r_2 showed their practical application in metabolic engineering. This study provides new candidate promoters for metabolic engineering and synthetic biology, and fills the gap due to the absence of well-characterized promoters in *Ketogulonicigenium* species.

## Materials and Methods

### Strains and Plasmids

All the strains and plasmids used in this study were listed in Table S1. The plasmid pBBR1MCS-2 [26] was used for gene expression in host strains, and *E. coli* WM3064 was used as a donor strain in conjugation with *K. robustum* SPU_B003, or *K. vulgare* SPU B805 or *P. denitrificans* PD1222.

### Culture Medium and Growth Condition

The broth medium for *K. robustum* SPU_B003 contained 20 g/l corn steep liquor, 10 g/l peptone, 10 g/l sorbitol, 10 g/l mannitol and 10 g/l CaCO_3_ (pH 6.5). The plate medium for *K. robustum* SPU_B003 contained 3 g/l yeast extract, 3 g/l beef extract, 3 g/l corn steep liquor, 10 g/l peptone, 1 g/l MgSO_4_, 5 g/l sorbose, 1 g/l CaCO_3_ and 20 g/l agar (pH 6.5). The fermentation medium for *K. robustum* SPU_B003 contained 20 g/l corn steep liquor, 70 g/l L-sorbose, 1 g/l MgSO_4_, 0.04 g/l nicotinamide, 0.37 g/l calcium pantothenate, 0.168 g/l aminobenzoic acid, and 25 g/l CaCO_3_ (pH 7.0). The plate medium for *K. vulgare* SPU B805 contained 20 g/l L-sorbose, 1 g/l KH2PO_4_, 3 g/l yeast powder, 0.2 g/l MgSO_4_, 3 g/l beef extract, 1 g/l urea, 10 g/l peptone, 3 g/l corn steep liquor and 20 g/l agar (pH 6.8). The broth medium for *K. vulgare* SPU B805 contained 20 g/l L-sorbose, 1 g/l KH2PO_4_, 3 g/l yeast powder, 0.2 g/l MgSO_4_, 12 g/l urea, and 10 g/l corn steep liquor (pH 6.8). Both *K. robustum* SPU_B003 and *K. vulgare* SPU B805 were cultured at 30°C. *P. denitrificans* PD1222 was cultured in LB medium at 30°C. *P. putida* KT2440, *B. licheniformis* and *R. ornithinolytica* were all cultivated in LB medium at 37°C. Finally, 30 μg/ml kanamycin was supplemented to the medium when the cultured strain harbored plasmid.

### Selection and Prediction of Putative Promoters

The housekeeping genes of *K. robustum* SPU_B003 were selected by transcriptional level of transcriptome and the UTRs were selected by genome sequence, and then subjected to BDGP (Berkeley Drosophila Genome Project, http://www.fruitfly.org/seq_tools/promoter.html) and BPROM (Prediction of bacterial promoters, http://www.softberry.com/berry.phtml?topic=bprom&group=programs&subgroup=gfindb) [27] to predict the possibility of promoters and the -10 and -35 box. The sigma factor of each putative promoter was recognized and predicted through BacPP (Bacterial promoter prediction, http://www.bacpp.bioinfoucs.com/home) [28]. The conservative region analysis was completed by Weblogo 3.0 (http://weblogo.threeplusone.com/create.cgi).

### DNA Manipulation

The putative promoters were amplified from genomic DNA of *K. robustum* SPU_B003. The *gfp* gene was cloned from the plasmid pX551-*gfp* and used as a reporter. After purification by a DNA cleanup kit, the promoter and *gfp* fragments were fused to obtain P_*gfp* by overlap PCR. The P_*gfp* fragment and expression plasmid pBBR1MCS-2 were digested with restriction endonuclease EcoRI and BamHI, then the digested plasmid and P_*gfp* fragment were ligated using T4 DNA ligase to obtain the recombinant plasmid pBBR-P_*gfp*.

The *minCD* genes were amplified by PCR from the genome of *P. putida* KT2440. The promoters and *minCD* genes were fused by overlap PCR using P-F and *minCD*-R primers to generate P_*minCD*. Then, fragment P_*minCD* and plasmid pBBR1MCS-2 were digested with HindIII and XbaI, and ligated to obtain pBBR-P_*minCD*. The primers used in this study were listed in Table S2.

The recombinant plasmids were transferred from *E. coli* WM3064 to *K. robustum* SPU_B003, *K. vulgare* SPU B805 and *P. denitrificans* PD1222 by conjugation [29] with optimization as follows: the donor strain and recipient strain were cultured in fresh medium until they reached logarithmic phase. The cells were harvested by centrifugation at 6,000 g for 10 min and washed twice with 0.9 % NaCl, and then the cell pellets were resuspended in 200 μl 0.9 % NaCl. The donor and recipient strains were then mixed together by vortexing. A piece of nitrocellulose membrane was plated on the plate medium or LB agar medium, and then the mixture was dropped on nitrocellulose membrane. After incubation for 12 h, the cells were collected and resuspended in broth medium for *K. robustum* SPU_B003 and *K. vulgare* SPU B805, and LB medium for *P. denitrificans* PD1222 for 3 h incubation at 30°C. Finally, 200 μl of the suspension was spread on the medium plates with 30 μg/ml kanamycin and incubated at 30°C for 2-3 days. The transformation of the recombinant plasmids to *P. putida* KT2440, *B. licheniformis* and *R. ornithinolytica* was conducted by electroporation [30].

The sorbose dehydrogenase gene (*sdh*) and *cyt*ochrome *c551* (*cyt* *c551*) were cloned from the genome DNA of *K. vulgare* SPU B805, fused with P_k.r_1 and P_k.r_2 to obtain P_k.r_1_*sdh* and P_k.r_2_*cyt* *c551* by overlap PCR, and finally ligated to the pBBR1MCS-2 vector to generate pBBR-P_k.r_1_*sdh*, pBBR-P_k.r_2_*cyt* *c551* and pBBR-P_k.r_1_*sdh*-P_k.r_2_*cyt* *c551* after digestion by restriction endonuclease.

### GFP Fluorescence Determination

The recombinant strains were cultured in seed medium or LB medium supplemented with 10 mg/ml kanamycin for 24 h, then harvested by centrifugation at 6,000 g for 10 min and washed twice with PBS buffer. The whole cell fluorescence of GFP (RFU/OD_600_) was determined by using a fluorescence microplate reader (Infinite M200 Pro, Tecan, Switzerland) at 485 nm for excitation wavelength and 535 nm for emission wavelength. At the same time, the cell density was measured at 600 nm, and then all samples were diluted to OD_600_ of 1.0 before the fluorescence was determined. The fluorescence of wild-type strain was subtracted as background from the overall fluorescence of the recombinant strains.

### Quantitative Real-Time PCR

The cells cultured for 24 h were collected by centrifugation at 10,000 g for 10 min at 4°C. Then, the total RNA was extracted with a Bacteria RNA Extraction Kit (Vazyma Biotech Co. Ltd., China). Quantitative real-time PCR was performed by using GoTaq qPCR Master Mix on an Mx3000P (Agilent Technologies, Inc, USA) with a total volume of 20 μl containing 2 μl cDNA, and *gfp*-q-F and *gfp*-q-R as primers for the amplification of *gfp* or 16s rRNA-q-F and 16s rRNA-q-R for internal standard. The samples were initially denatured at 95°C for 2 min, and subsequently denatured at 95°C for 15 s and incubated at 60°C for 1 min to allow the primers to anneal to the template, which were repeated for 40 cycles. The 16S rRNA gene was selected as the internal standard and the designed primers were listed in Table S2.

### Expression of SDH and Cytochrome *c551*

The engineering strain was collected by centrifugation at 10,000 g for 10 min at 4°C, and the pellet was resuspended in PBS buffer. Then, the suspension was disrupted by sonication (10 min for 3-s pulses, leaving 5 s between each pulse) and followed by centrifugation at 10,000 g for 10 min at 4°C. The expression of SDH and *cyt* *c551* was detected by SDS-PAGE using a 12% running gel.

### Morphological Observation of Recombinant Strains

Morphological observation was performed by microscope with DIC mode (Olympus BX53F, Japan).

### Analysis of Cell Density and 2-KGA Concentration

The cell density of *K. robustum* SPU_B003 was calculated by the CFUs in the plate medium.

The concentration of 2-KGA in fermentation broth was measured by high-performance liquid chromatography (HPLC) (Shimadzu Corporation, Japan) using an amino column (SUPELCOSIL LC-NH2, 25 cm × 4.6 mm, 5 μm, Sigma, USA) at 210 nm. The mobile phase used was acetonitrile-KH_2_PO_4_ (5%/95%, v/v) with a flow rate of 0.6 ml/min [12]. A series of 2-KGA standard solutions with different concentrations were prepared for HPLC detection. The standard curve between 2-KGA concentration and peak area was drawn to calculate the content of 2-KGA in the fermentation broth.

## Results

### In Silico Prediction of the Putative Constitutive Promoters

To facilitate the screening of promoters at different levels, samples at different fermentation time points were taken for transcriptome detection (not published). A total of 41 housekeeping genes were selected according to their transcriptional levels, which were represented by the FPKM value (fragments per kilobase of transcript per million fragments sequenced, represented the quantity of gene transcript) [31] (Table S3). The UTRs of the 41 housekeeping genes of *K. robustum* SPU_B003 were selected according to the genome sequence (NCBI accession number CP019937), and then subjected to BDGP to predict the possibility of being promoters. The result showed only four of them had a relatively high score with great ability to be promoters (Table 1), so the DNA sequences of these putative promoters were extracted for further study (Table S4) and deposited in the GenBank database with the accession number of MH919400-919403.

### Promoter Sequence Analysis

**Fig. 1:**
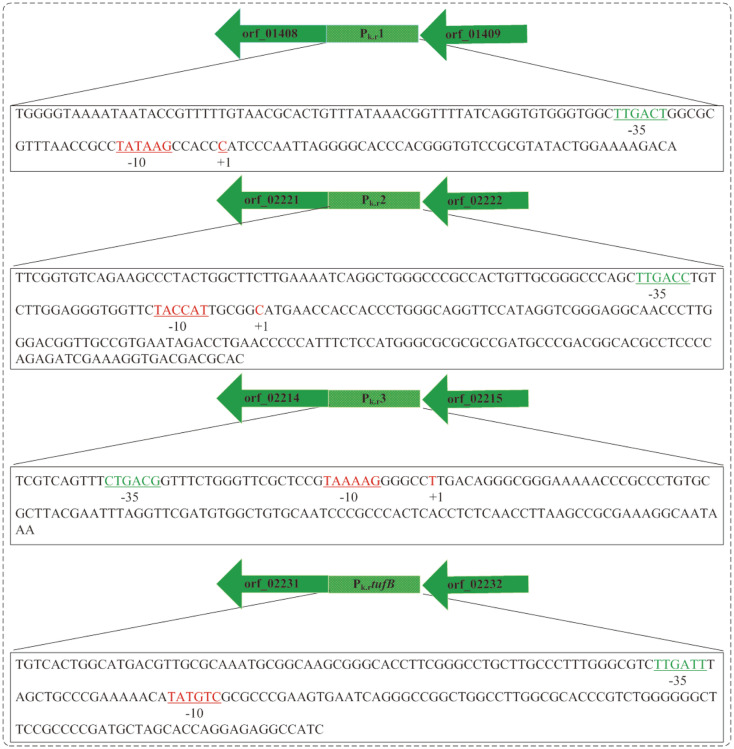
Schematic representation of the *K. robustum* SPU_B003 putative promoter.

**Fig. 2:**
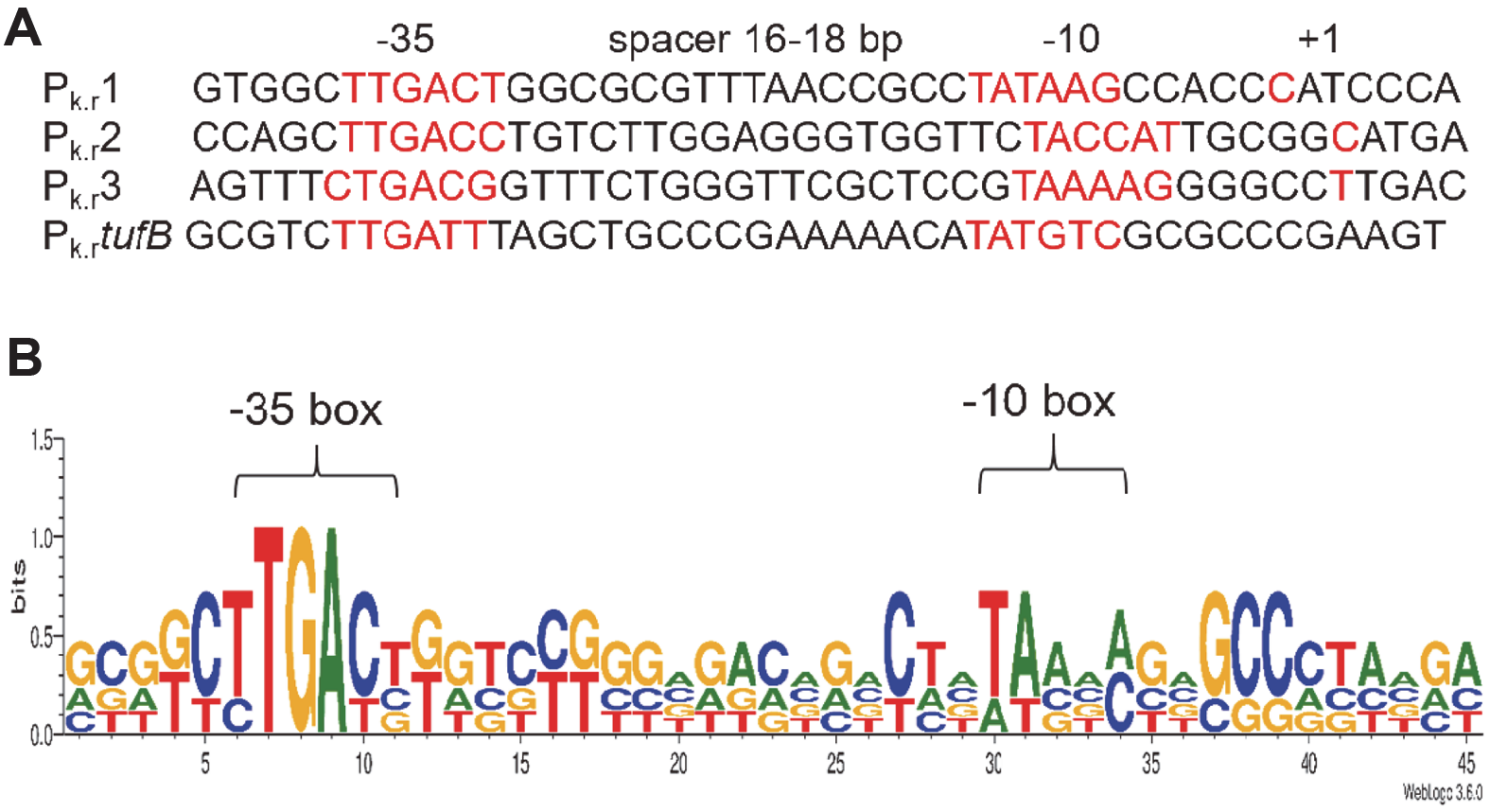
The consensus Logo of the four promoters.

Each of the four promoters contains putative transcription start sites, -10 and -35 box, and the spacing length between -10 and -35 box is 16-18 bp, which conforms to the basic characteristics of promoters (Figs. 1 and 2A). The specific σ factor analysis by BacPP website indicated that the putative -10 and -35 boxes of P_k.r_1 and P_k.r_2 were similar to the consensus recognition sequence of *E. coli* σ^70^ (-10 box TATAAT, -35 box TTGACA) and that of P_k.r_3 and P_k.r_*tufB* closely resembled the heat shock protein σ^32^ (-10 box CTCTAWWWW, -35 box YTKRWWW, where Y, W, K and R stand for C or T, A or T, G or T, A or G, respectively) [28]. Furthermore, the promoter sequence logos were created using WebLogo, and the result showed the -10 and -35 regions between the four promoters were not very conservative, indicating they belonged to different promoters (Fig. 2B). Moreover, the identity of these promoters with commonly used promoters of *E. coli* (*tufB*, lac, lacUV5) [32], *K. vulgare* (P*sdh*, Psndh) [33] and *P. putida* KT2440 (JE111411) [32] were performed, and the result showed the nucleotide similarity was only 12.62%, indicating these putative promoters were new genetic elements that are significantly different from the previous ones (Fig. S1).

### The Initiated Intensity of the Four Putative Promoters in *K. robustum* SPU_B003

**Fig. 3:**
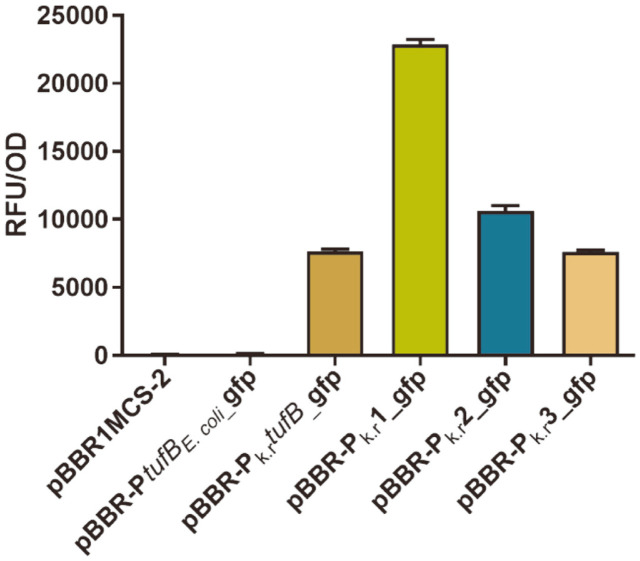
The whole cell relative fluorescence intensity (RFU/OD) detection of recombinant *K. robustum* SPU_B003 strains. Data represent the mean ± SD of 3 replicates.

Putative promoters with high FPKM values and high scores were amplified from genome by PCR and inserted into expression vector pBBR1MCS-2 with *gfp* as a reporter for activity identification, and the commonly used *tufB* promoter of *E. coli* [34] was selected as a control. As observed by fluorescence microscope, the GFP fluorescence signal was minimal in the recombinant strain harboring blank plasmid pBBR1MCS-2, which indicated that the plasmid pBBR1MCS-2 had no effect on the observation of fluorescence, and can therefore be used as expression vector to detect promoter activity in *K. robustum* SPU_B003 (Fig. S2A). The strain containing promoter P*tufB_E.coli_* showed no obvious fluorescence, indicating the promoter couldn’t initiate the expression of *gfp* in *K. robustum* SPU_B003 because of its species specificity (Fig. S2B). As for the four putative promoters, P_k.r_*tufB*, P_k.r_1, P_k.r_2, and P_k.r_3, the fluorescence intensity was significantly higher than that of control, especial promoter P_k.r_1, indicating all of them had the ability to drive gene expression and had great value for further study (Figs. S2C-S2F). Therefore, the intensity of promoters was determined through whole cell fluorescence (RFU/OD) by a fluorescence microplate reader. As shown in Fig. 3, the whole cell fluorescence of promoter P_k.r_1 was 22,187 ± 664.6, exhibiting the strongest fluorescence intensity, followed by promoter P_k.r_2 (RFU/OD 10,617 ± 697.8), P_k.r_*tufB* (RFU/OD 7,655 ± 294.7) and P_k.r_3 (RFU/OD 7,590 ± 287.5), which were in accordance with the results observed by fluorescence microscope (Fig. S2). Furthermore, the relative transcriptional level of *gfp* gene was detected by RT-qPCR, and the results showed that the transcriptional level of *gfp* initiated by promoter P_k.r_1 was the highest, followed by P_k.r_2, and the relative transcriptional level of promoter P_k.r_3 was basically the same as that of P_k.r_*tufB* (Fig. S3). The relative transcriptional level of *gfp* showed the same trend as the fluorescence detection results, indicating that the whole cell fluorescence assays can be used to measure promoter strength, and so it was adopted for subsequent cross-species verification. The above results indicated that these intronic promoters were functioning well in initiating *gfp* expression in *K. robustum* SPU_B003, and therefore have great potential in rational fine-tuning of protein expression for achieving increased yields in *K. robustum* SPU_B003.

### Application of the Promoters in Cross-Species Microorganism

To further evaluate the performance of the above promoters in other bacteria, the recombinant plasmids with different promoters were firstly transformed into *K. vulgare* SPU B805, another species of *Ketogulonicigenium*, which can produce 2-KGA accompanied by B. megaterium. The whole cell fluorescence showed that all of the four promoters could initiate the *gfp* gene expression in the order of P_k.r_1 (RFU/OD 21,854 ± 1,139), P_k.r_2 (RFU/OD 9,950 ± 530.2), P_k.r_3 (RFU/OD 7,390 ± 144.9), P_k.r_*tufB* (RFU/OD 7,422 ± 229.8) (Fig. S4), which was similar to that in *K. robustum* SPU_B003, indicating these promoters functioned well in different *Ketogulonicigenium* species. These promoters filled the gaps of the deficiency of specific promoters to realize rational design and optimization of metabolic pathway in *Ketogulonicigenium* species.

**Fig. 4:**
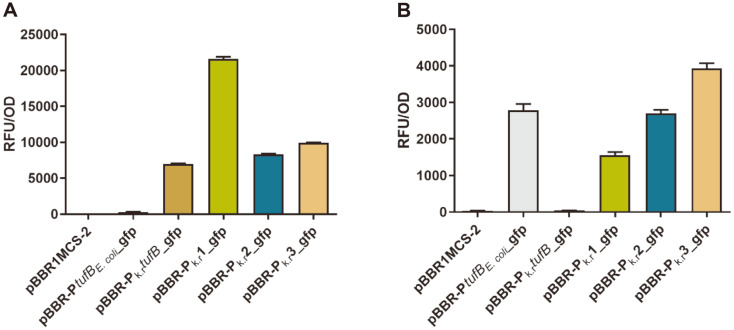
The whole cell relative fluorescence intensity (RFU/OD) detection of different recombinant *P. putida* KT2440 and *P. denitrificans* PD1222. (**A**) and (**B**) were recombinant *P. putida* KT2440 and *P. denitrificans* PD1222 harbored different recombinant plasmids. Data represent the mean ± SD of 3 replicates.

*P. putida* KT2440 and *P. denitrificans* PD1222, being commonly used strains for genetic editing of biochemical network to produce target compounds of interest, were selected for further cross-species studies. As shown in Figs. 4A and S5, in *P. putida* KT2440, promoter P_k.r_1 showed the strongest fluorescence intensity with the RFU/OD of 21,608.4 ± 190.3, which was consistent with that in *K. robustum* SPU_B003 and *K. vulgare* SPU B805, indicating P_k.r_1 is of robust, high-efficiency and cross-species characteristics. Next in order of fluorescence intensity were P_k.r_3 (RFU/OD 9,929 ± 87.44), P_k.r_2 (RFU/OD 8,317 ± 194.7) and finally P_k.r_*tufB* (RFU/OD 6,994 ± 126.1). Compared to the promoters reported by Elmore *et al*. [32], the strength of P_k.r_1 and P_k.r_3 were significantly higher than the strongest promoter JE111411 (RFU/OD was 8,450 ± 3,950), and also higher than the control promoter PlacUV5 (RFU/OD was 8,472 ± 1,679) and Plac (RFU/OD was 1,131 ± 688), and even the weakest promoter P_k.r_*tufB* was stronger than the Plac and JE121511 (RFU/OD was 623 ± 321). Besides, the strength of both P_k.r_1 in *Ketogulonicigenium* sp. and *P. putida* KT2440 was also higher than that of gHp0169 in *G. oxydans* (RFU/OD was 16,000) [9]. These data demonstrated the four identified promoters were all of higher ability in rational regulating target gene expression and these promoters enriched the genetic elements for fine-tuning protein expression in *P. putida*.

In *P. denitrificans* PD1222, promoter P_k.r_3 (RFU/OD 3,934 ± 238.1), P_k.r_2 (RFU/OD 2,703 ± 174.0), P_k.r_1 (RFU/OD 1,558 ± 145.1) were functional, and the fluorescence intensities decreased gradually in the above order (Figs. 4B and S6). Surprisingly, the *E. coli*_*tufB* promoter (RFU/OD 2,786 ± 301.0) displayed a relatively stronger fluorescence even than the other three promoters except for promoter P_k.r_3. These results indicated that promoter performance can be influenced by host genetic background.

In addition, more bacterial strains, *B. licheniformis* and *R. ornithinolytica*, were also selected to conduct the promoter cross-species study. *B. licheniformis* is a well-characterized bacterium used in a variety of productions, and *R. ornithinolytica* represents a plant rhizosphere strain that can be used for the study of rhizosphere soil microorganisms [35]. Expectedly, promoter P_k.r_1 showed the highest fluorescent strength compared to the other promoters in *B. licheniformis*, indicating its broad suitability across species. However, different from P_k.r_1, promoter P_k.r_2 showed the strongest activity in *R. ornithinolytica* (Fig. S7). These results suggested that different promoters have different host adaptations: some promoters appeared to be robust in cross-context bacteria, while others showed different efficiencies in different host genetic backgrounds.

The strength of the strongest promoter, P_k.r_1, was 2.9-fold higher than that of the weakest promoter, P_k.r_3, in *K. robustum* SPU_B003 and *K. vulgare* SPU B805, and 3.1-fold higher (the strongest promoter P_k.r_1 compared to promoter P_k.r_*tufB* ) in *P. putida* KT2440 and 98-fold higher (the strongest promoter P_k.r_3 compared to promoter P_k.r_*tufB* ) in *P. denitrificans* PD1222. These results demonstrated their potential as alternative promoters and broadened the selection for rational design and accurate regulation of metabolic pathway. Moreover, the application in cross-species bacteria with different genetic backgrounds has also enriched the usable range of these promoters.

### Validation Experiments in Gene Engineering Strains Using Novel Promoters

To demonstrate whether these novel promoters could be practically used in metabolic engineering, P_k.r_1 and P_k.r_2 were selected to express phosphotransketase (*xfp*) and phosphotransacetylase (*pta*) heterologously to regulate the metabolic flow in the direction of reducing carbon loss in *K. robustum* SPU_B003. The results showed the acetyl-CoA level of recombinant strain was increased by approximately 2.4-fold and the yield of target product 2-KGA was enhanced by 22.27% [12], indicating the identified promoters have been successfully applied to regulate the target gene expression of the specific pathway in synthetic biology.

Further, *P. putida* KT2440 was selected to overexpress the *minCD* cassette initiated by the four promoters to validate their usability in genetic engineering across species. Cell division in bacteria is regulated by the Min system, which consists of three genes, *minC*, *minD*, and *minE*. MinC acts an inhibitor of FtsZ polymerization that is activated by MinD and regulated by MinE. FtsZ is the most conserved component of the bacterial cell division machinery [36], and assembles into a Z ring at the mid-cell and results in the formation of daughter cells. Overexpression of *minC* and *minD* results in the inhibition of cell division at the potential division sites and induces long non-septate filaments of rod-shaped strains [37] and swelling of coccoid bacteria [38]. As expected, the cells of *P. putida* KT2440 carrying pBBR-P-*minCD* became long filaments (Figs. S8C-S8F), indicating these promoters successfully initiated the expression of the *minCD* genes and then resulted in the prevention of cell division in the engineering strains; whereas those of cells carrying blank vector pBBR1MCS-2 showed the same bacilliform shapes as the wild type (Figs. S8A and S8B). These results suggested that the isolated promoters can indeed activate target gene expression and achieve the desired effect in metabolic engineering.

### Expression of Sorbose Dehydrogenase and *cyt* *c551* Using P_k.r_1 and P_k.r_2 Promoter

To investigate whether the promoters can be used to enhance the 2-KGA yield, promoters P_k.r_1 and P_k.r_2 were applied to express sorbose dehydrogenase (SDH) or *cyt*ochrome *c551* (*cyt* *c551*), the key enzyme for the oxidization of L-sorbose to 2-KGA and respiratory chain in *K. robustum* SPU_B003. An orthogonality test of the promoters and genes showed P_k.r_1 for *sdh* and P_k.r_2 for *cyt* *c551* were the best combination. Moreover, the SDS-PAGE showed that the genes *sdh* and *cyt* *c551* were expressed in the engineered strain harboring pBBR-P_k.r_1_*sdh*-P_k.r_2_*cyt* *c551* plasmid (Fig. S9).

In the process of L-sorbose oxidization to 2-KGA by sorbose dehydrogenase, the electron released in the dehydrogenation reaction entered into the respiratory chain electron transfer system through coenzyme PQQ, which means that the electron transport chain of the pyrroloquinoline quinone-dependent dehydrogenases (SDH) was closely coupled with respiratory chain electron transfer system [39]. The functional deficiency or functional insufficiency of the pyrroloquinoline quinone-dependent dehydrogenases - respiratory chain electron transfer system resulted in the electron emission in the respiratory chain and therefore ROS (Reactive Oxygen Stress) occurrence in the fermentation of *K. robustum* SPU_B003, which finally affected the growth of this strain. Therefore, overexpression of *sdh* resulted in the electron leakage of respiratory chain, which imposed a burden on the growth of *K. robustum* SPU_B003. Overexpression of *cyt* *c551* can increase the electron transfer efficiency to some extent and reduce the pressure caused by electron leakage in metabolism. Furthermore, the simultaneous overexpression of *sdh* and *cyt* *c551* alleviated the pressure of respiratory chain electron transfer system and contributed to the production of 2-KGA, which was partly be converted into idonate by gluconate 2-dehydrogenase (GA2DH), and subsequently entered the pentose phosphate pathway for energy and biomass production [13].

**Fig. 5:**
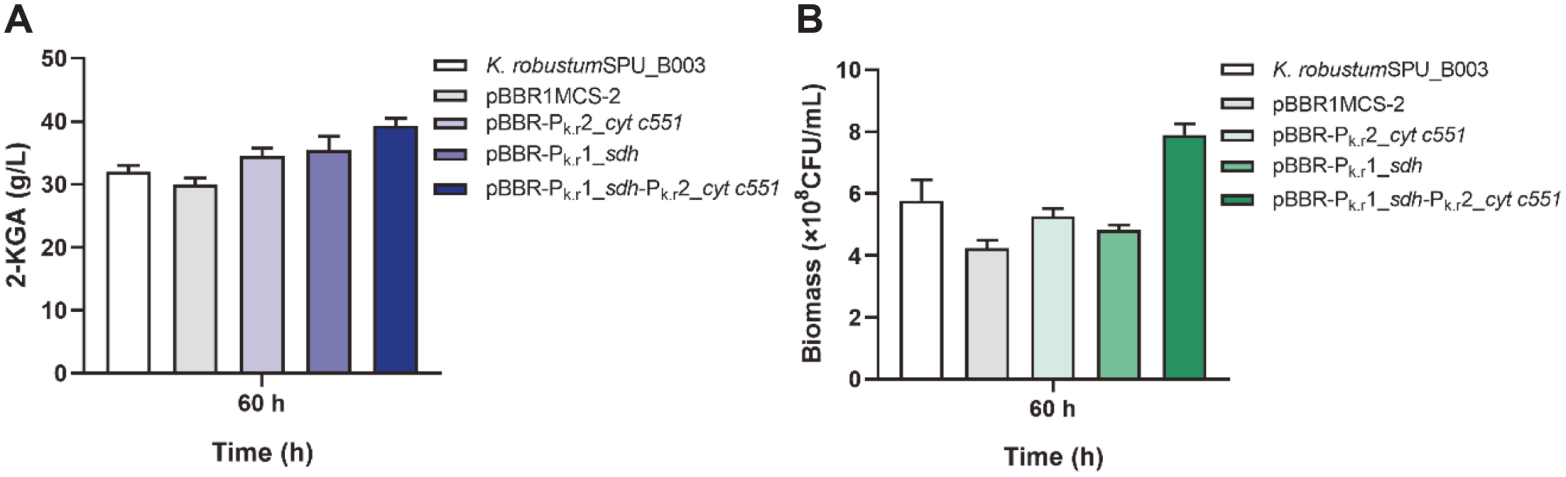
The 2-KGA yield and biomass in the fermentation. (**A**) The 2-KGA yield of different recombinant strains at 60 h; (**B**) the biomass of different recombinant strains at 60 h.

The above analysis was confirmed by the results that the 2-KGA yield of the recombinant strains containing the plasmid pBBR-P_k.r_1_*sdh*-P_k.r_2_*cyt* *c551* were 22.75% higher than that of wild-type *K. robustum* SPU_B003 (32.01 g/l) at 60 h (Fig. 5A), and the biomass of the recombinant strain was also higher than the wild type (Fig. 5B), which indicated the overexpression of 2-KGA biosynthesis pathway and the respiratory chain was helpful in increasing the yield of 2-KGA. This means that the newly identified promoters will have practical applications in the metabolic engineering practices.

## Discussion

The critical step of regulating gene expression in the metabolic pathway is to control the metabolic flux towards the target biosynthetic pathway in cells. But in fact, no matter the modification of the original metabolic pathway or the introduction of heterologous biosynthetic pathway, it always disturbs the native metabolism and results in the unbalance of native metabolic flux. Because of promoters’ substantial influence on gene expression, promoter engineering is considered to be one of the most effective strategies to fine-tune gene transcription. Although many promoters have been identified and reported in different microorganisms, their activities often changed unexpectedly when the promoters were transferred from one strain/species to another [8, 33]. Host cells often interfere with the activities of promoters and result in unpredictable changes in expression level [8]. Deciphering the interactions between promoters and hosts is always challenging, and solving the host-interference problems is often difficult. Therefore, development of a set of robust, efficient and specific promoters for fine-tuning the expression of genes is still an urgent task in metabolic engineering.

In this study, we attempted to address the dearth of promoters for genetic and metabolic engineering and characterize endogenous promoters across a broad spectrum of bacterial chassis to control expression. *Ketogulonicigenium* sp., a 2-KGA-producing strain, has been extensively studied in genetics and genetic engineering to improve strain traits and enhance 2-KGA yield; however, the limited availability of endogenous promoters is often a bottleneck to precise transcriptional regulation. This aroused our avid desires for development of high-performance promoters to meet the requirements for rational design and accurate regulation of target metabolic network. So, we predicted the house-keeping promoters of *K. robustum* SPU_B003 based on the transcriptome data and genome sequence, and then identified their activity with *gfp* as reporter in *K. robustum* SPU_B003. Finally, we transformed them into bacteria strains with a significantly different genetic context to address their availability across species. The results showed that the four promoters functioned well in *K. robustum* SPU_B003, *K. vulgare* SPU B805, *P. denitrificans* PD1222 and *P. putida* KT2440. Promoter P_k.r_1 showed the strongest gene expression capability in *K. robustum* SPU_B003, *K. vulgare* SPU B805, and *P. putida* KT2440, implying its high efficiency, robust and cross-species activity. While in *P. denitrificans* PD1222, the four promoters showed a relatively lower activity in initiating gene expression. Additionally, we further evaluated the strength of these promoters in two other bacteria, *B. licheniformis* and *R. ornithinolytica* (Fig. S7). Promoter P_k.r_2 showed a relatively strong ability in initating *gfp* expression in *R. ornithinolytica* but rather weak activity in *B. licheniformis*. These observations demonstrated that different promoters have different effective host ranges. Some promoters (P_k.r_2, P_k.r_3, P_k.r_*tufB*) are strongly affected by the host context, and therefore have a relatively narrow host range. However, promoter P_k.r_1 appeared to be robust regardless of the genetic background, indicating that it has wide applicability in cross-species bacteria. Besides, the comparison of P_k.r_1 with promoter *gHp0169*, PlacUV5, Plac and JE121511 further demonstrated its strong intensity in regulating gene expression in metabolic engineering.

To control the metabolic flux towards the direction of reducing carbon loss in *K. robustum* SPU_B003, promoters P_k.r_1 and P_k.r_2 were selected to construct heterologous XFP-PTA pathway. The increased acetyl-CoA level and 2-KGA yield of recombinant strain indicated that the promoters were capable of regulating the expression of target genes towards specific pathway and the identified promoters were able to be used to control the metabolic flux in synthetic biology. In addition, the morphological changes of *P. putida* KT2440 from rod-shaped strains to long non-septate filaments also demonstrated the identified promoters initiated the target gene expression, which indicated these promoters practically played a role in cross-species bacteria. Furthermore, in order to enhance the 2-KGA yield of *K. robustum* SPU_B003, *sdh* and *cyt* *c551* genes were overexpressed by P_k.r_1 and P_k.r_2, and the results showed a 22.75% and 10.81% enhancement compared to wild-type strain. As more experiments are conducted, these promoters will be used to regulate expression of more genes in the balance of metabolic flux for pathway optimization, and they will also be applied in more hosts to design and generate desired microbial cell factories for industrial applications.

In summary, this study provided a wide dynamic range of promoters available for gene expression in cross-species with different genetic contexts, and in the meantime enriched the promoter pool to precisely control gene expression and balance the flux to increase the yield of target products in metabolic engineering. This research serves as a good example of applying bioinformatics to find new biological parts for gene engineering to reduce the interference of host background. It is expected these promoters will be broadly used for the fine-tuning of flux in metabolic engineering and synthetic biology in many other microorganisms. Further saturation mutagenesis inside the promoter core region will increase the diversity promoter library for gene expression.

## Supplemental Materials

Supplementary data for this paper are available on-line only at http://jmb.or.kr.

## Figures and Tables

**Table 1:** The information on four putative constitutive promoters.

Promoter	Gene locus tag	Gene product	FPKM	Score
P_k.r_1	BVG79_01408	50S ribosomal protein L13	600.279	0.98
P_k.r_2	BVG79_02221	DNA-directed RNA polymerase subunit beta	722.0759	0.88
P_k.r_3	BVG79_02214	30S ribosomal protein S10	464.4049	0.9
P_k.r_*tufB*	BVG79_02215	Elongation factor Tu	238.8473	0.75
