# Supplementary material for: Identification and Validation of Four Novel Promoters for Gene Engineering with Broad Suitability across Species
Source: J Microbiol Biotechnol. 2021 Jun 29;31(8):1154–62. doi: 10.4014/jmb.2103.03049 (PMC9706022; doi:10.4014/jmb.2103.03049)
Supplement: Supplementary file 1 [file jmb-31-8-1154-supple.pdf]

J. Microbiol. Biotechnol.
https://doi.org/10.4014/jmb.2103.03049

Table S1. List of strains and plasmids used in this study

Names	Descriptions	Sources
**Strains**		
K. robustum SPU_B003	2-KGA-producing strain	This lab [1]
E. coli WM3064	RP4 (*tra*) in chromosome, DAP$^-$	This lab [1]
P. putida KT2440	Wild type	This study
P. denitrificans PD1222	Wild type	This study
K. vulgare SPU B805	2-KGA-producing strain, need the accompany of *Bacillus megatherium* SPU B806	This lab [2]
B. licheniformis	Wild type	This study
R. ornithinolytica	Wild type	This study
**Plasmids**		
pBBR1MCS-2	Broad host plasmid	This lab [1]
pX551-*gfp*	Plasmid carries *gfp* gene	This lab [1]
pBBR-P*tufB*$_{E.coli}$_*gfp*	Recombinant plasmid carries *gfp* gene initiated by *E. coli_tufB* promoter	This study
pBBR-P$_{k.r}$*tufB_gfp*	Recombinant plasmid carries *gfp* gene initiated by *tufB* promoter of *K. robustum* SPU_B003	This study
pBBR-P$_{k.r}$1_*gfp*	Recombinant plasmid carries *gfp* gene initiated by P$_{k.r}$1 promoter	This study
pBBR-P$_{k.r}$2_*gfp*	Recombinant plasmid carries *gfp* gene initiated by P$_{k.r}$2 promoter	This study

pBBR-$P_{k.r}$3_*gfp*	Recombinant plasmid carries *gfp* gene initiated by $P_{k.r}$3 promoter	This study
pBBR-$P_{k.r}$*tufB_minCD*	Recombinant plasmid carries *minCD* gene initiated by SPU_B003_*tufB* promoter for morphology study	This study
pBBR-$P_{k.r}$1_*minCD*	Recombinant plasmid carries *minCD* gene initiated by $P_{k.r}$1 promoter for morphology study	This study
pBBR-$P_{k.r}$2_*minCD*	Recombinant plasmid carries *minCD* gene initiated by $P_{k.r}$2 promoter for morphology study	This study
pBBR-$P_{k.r}$3_*minCD*	Recombinant plasmid carries *minCD* gene initiated by $P_{k.r}$3 promoter for morphology study	This study

2

Table S2. List of primers used in this study

Names	Sequence (5' to 3')	Restriction sites
$P_{k.r}$1-F	ctgccgGAATTCTGGGGTAAAATAATACCGTTTTTGTAACGCAC	*Eco*R I
$P_{k.r}$1-R	tgaaaagttcttctcctttacgcatTGTCTTTTCCAGTATACGCGGACAC	—
$P_{k.r}$2-F	ctgccgGAATTCTTCGGTGTCAGAAGCCCTACTG	*Eco*R I
$P_{k.r}$2-R	tgaaaagttcttctcctttacgcatGTGCGTCGTCACCTTTCGATC	—
$P_{k.r}$3-F	ctgccgGAATTCTCGTCAGTTTCTGACGGTTTCTGG	*Eco*R I
$P_{k.r}$3-R	tgaaaagttcttctcctttacgcatTTTATTGCCTTTCGCGGCTTAAGG	—
P*tufB*$_{E.coli}$-F	ctgccgGAATTCCAGGAGCGCATTGTTGAGCACAATG	*Eco*R I
P*tufB*$_{E.coli}$-R	tgaaaagttcttctcctttacgcatCGATTGTCCCTCTAAGACACGG	—
$P_{k.r}$*tufB*-F	ctgccgGAATTCGGCCCGTCACAAGACGGTC	*Eco*R I
$P_{k.r}$*tufB-R*	tgaaaagttcttctcctttacgcatGGGTCTCTCTTTTCAGGAATTGTC	—
gfp-F	atgcgtaaaggagaagaacttttcac	—
gfp-R	cgacgcGGATCCaaaaaaaaccccgccctgtcaggggcggggttttttttTTATTTGTAT AGTTCATCCATGC	*Bam*H I
$P_{k.r}$*tufB*-F2	cccAAGCTTGGCCCGTCACAAGACGGTC	*Hin*d III
$P_{k.r}$*tufB*-R2	ggttgcccgcaacatgcacGGGTCTCTCTTTTCAGGAATTGTC	—
$P_{k.r}$1-F2	cccAAGCTTTGGGGTAAAATAATACCGTTTTTGTAACGCAC	*Hin*d III
$P_{k.r}$1-R2	ggttgcccgcaacatgcacTGTCTTTTCCAGTATACGCGGACAC	—
$P_{k.r}$2-F2	cccAAGCTTTTCGGTGTCAGAAGCCCTACTG	*Hin*d III
$P_{k.r}$2-R2	ggttgcccgcaacatgcacGTGCGTCGTCACCTTTCGATC	—
$P_{k.r}$3-F2	cccAAGCTTTCGTCAGTTTCTGACGGTTTCTGG	*Hin*d III

$P_{k.r}$3-R2	ggttgcccgcaacatgcacTTTATTGCCTTTCGCGGCTTAAGG	—
minCD-F	GTGCATGTTGCGGGCAACC	—
minCD-R	gcTCTAGATTATTTGCTCCCGAAAATGCGCTC	*Xba* I
$P_{k.r}$1-F3	gcTCTAGATGGGGTAAAATAATACCGTTTTTGTAACG	*Xba* I
$P_{k.r}$1-R3	TTGTCTTTTCCAGTATACGCGGAC	—
sdh-F	gtccgcgtatactggaaaagacaaATGAAACCGACTTCGCTGCTTTG	—
sdh-R	cgGGATCCTTATTGCGGCAGGGCGAAGAC	*Bam*H I
$P_{k.r}$2-F3	cgGAATTCTTCGGTGTCAGAAGCCCTACTG	*Eco*R I
$P_{k.r}$2-R3	GGTGCGTCGTCACCTTTCG	—
cyt c551-F	cgaaaggtgacgacgcaccATGAAAAACAAAACCACTCTGGG	—
cyt c551-R	cccAAGCTTTTTAGTTGGGGTTACGGCCCATC	*Hin*d III
gfp-q-F	TTACCAGACAACCATTACC	—
gfp-q-R	CAAACTCAAGAAGGACCAT	—
16s rRNA-q-F	ACAATGGTAGTGACAATGG	—
16s rRNA-q-R	GCGATTACTAGCGATTCC	—

Table S3. The information of the analyzed housekeeping gene

Gene name	Gene locus tag	FPKM	Gene name	Gene locus tag	FPKM
rpoB	BVG79_02221	722.0759	*rplP*	BVG79_02205	213.5253
rplM	BVG79_01408	600.279	*rplB*	BVG79_02209	209.0199
rpsJ	BVG79_02214	464.4049	*rplA*	BVG79_02224	208.731
rpsO	BVG79_02440	372.1691	*rplT*	BVG79_00228	200.8014
rplL	BVG79_02222	360.3161	*rplK*	BVG79_02225	188.2966
rplO	BVG79_02193	355.7171	*rpsK*	BVG79_02189	187.132
rpsU	BVG79_00234	351.7566	*rpsA*	BVG79_00523	171.6779
rpmD	BVG79_02194	341.524	*rpmF*	BVG79_01192	162.8542
rpsM	BVG79_02190	324.4464	*rpmG*	BVG79_02101	154.2556
rpmI	BVG79_00227	323.9393	*rpsG*	BVG79_02217	142.9593
rpsF	BVG79_00904	311.1587	*rpsH*	BVG79_02198	132.2903
rplD	BVG79_02212	298.7779	*rplV*	BVG79_02207	124.5978
rpsT	BVG79_02051	285.8584	*rpsR*	BVG79_00903	122.9474
rpmC	BVG79_02204	278.9984	*rpsQ*	BVG79_02203	119.6087
rpsS	BVG79_02208	266.385	*rpsN*	BVG79_02199	115.3851
rpsC	BVG79_02206	265.2982	*rplI*	BVG79_00902	105.7828
rpmA	BVG79_01560	256.3204	*rplE*	BVG79_02200	90.43514
rpmE	BVG79_00095	248.8347	*rplN*	BVG79_02202	87.15389
tufB	BVG79_02215	238.8473	*rplS*	BVG79_00096	83.13693

rplJ	BVG79_02223	235.5507	*rplX*	BVG79_02201	81.81784
rplQ	BVG79_02187	221.1563			

Table S4. The DNA sequences of the four putative promoters

Promoter name	Sequence (5’ to 3’)
$P_{k.r}1$	TGGGGTAAAATAATACCGTTTTTGTAACGCACTGTTTATAAACGGTTTTATCAG<br>GTGTGGGTGGCTTGACTGGCGCGTTTAACCGCCTATAAGCCACCCATCCCAAT<br>-35 -10 +1<br>TAGGGGCACCCACGGGTGTCCGCGTATACTGGAAAAGACA
$P_{k.r}2$	TTCGGTGTCAGAAGCCCTACTGGCTTCTTGAAAATCAGGCTGGGCCCGCCACT<br>GTTGCGGGCCCAGCTTGACCTGTCTTGGAGGGTGGTTCTACCATTGCGGCATG<br>-35 -10 +1<br>AACCACCACCCTGGGCAGGTTCCATAGGTCGGGAGGCAACCCTTGGGACGGTT<br>GCCGTGAATAGACCTGAACCCCCATTTCTCCATGGGCGCGCGCCGATGCCCGA<br>CGGCACGCCTCCCCAGAGATCGAAAGGTGACGACGCAC
$P_{k.r}3$	TCGTCAGTTTCTGACGGTTTCTGGGTTCGCTCCGTAAAAGGGGCCTTGACAGG<br>-35 -10 +1<br>GCGGGAAAAACCCGCCCTGTGCGCTTACGAATTTAGGTTCGATGTGGCTGTGC<br>AATCCCGCCCACTCACCTCTCAACCTTAAGCCGCGAAAGGCAATAAA
$P_{k.r}$*tufB*	TGTCACTGGCATGACGTTGCGCAAATGCGGCAAGCGGGCACCTTCGGGCCTGC<br>TTGCCCTTTGGGCGTCTTGATTTAGCTGCCCGAAAAACATATGTCGCGCCCGAA<br>-35 -10<br>GTGAATCAGGGCCGGCTGGCCTTGGCGCACCCGTCTGGGGGGCTTCCGCCCCG<br>ATGCTAGCACCAGGAGAGGCCATC

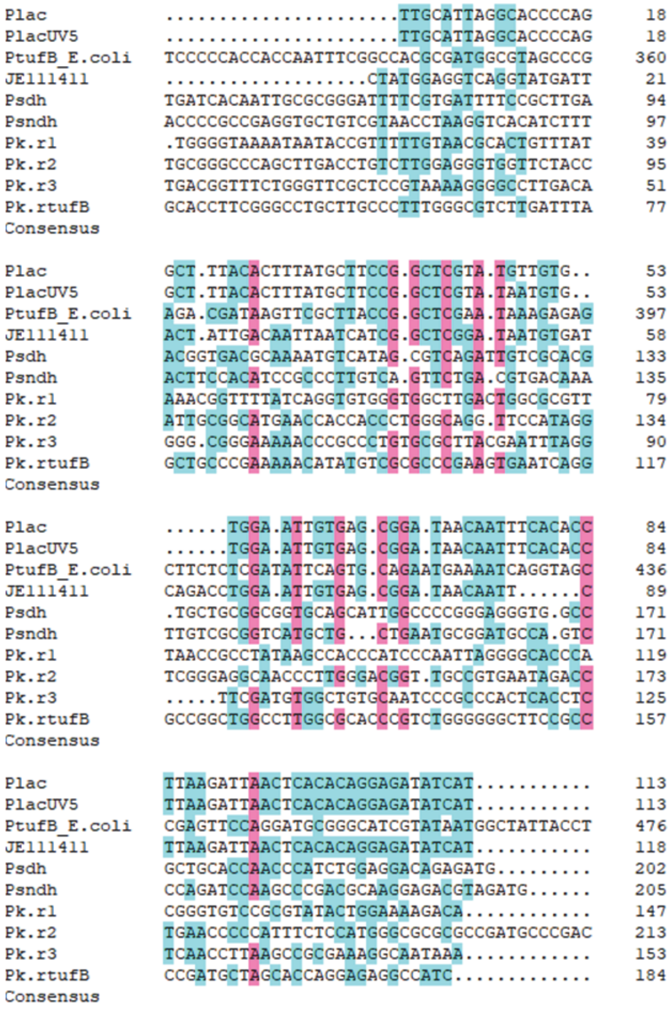

Figure S1. Alignment of the four putative promoters with commonly used promoters of *E. coli*, *K. vulgare* and *P. putida* KT2440

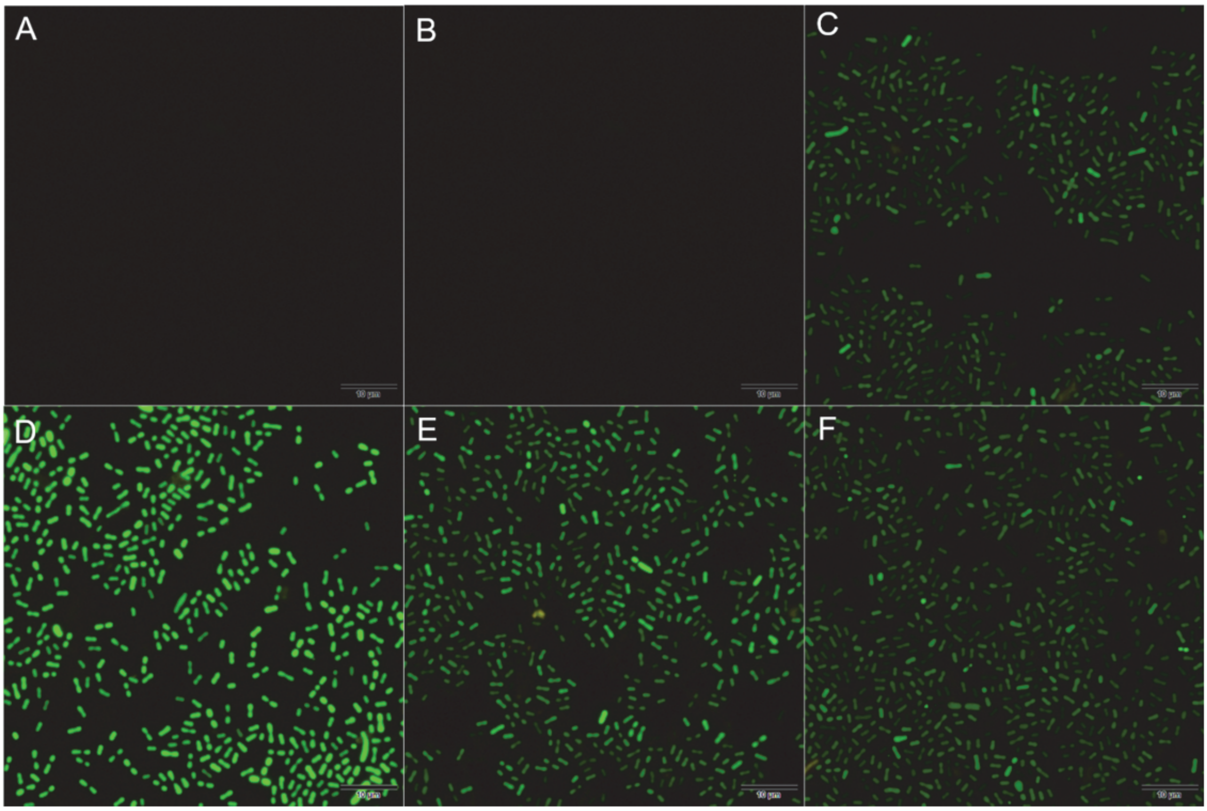

Figure S2. Fluorescence observation of recombinant *K. robustum* SPU_B003 strains. Strains harbored plasmids of (A) pBBR1MCS-2, (B) pBBR-P*tufBE. coli_gfp*, (C) pBBR-$P_{k.r}$*tufB_gfp*, (D) pBBR-$P_{k.r}$1*_gfp*, (E) pBBR-$P_{k.r}$2*_gfp* and (F) pBBR-$P_{k.r}$3*_gfp* were photographed with a fluorescence microscope.

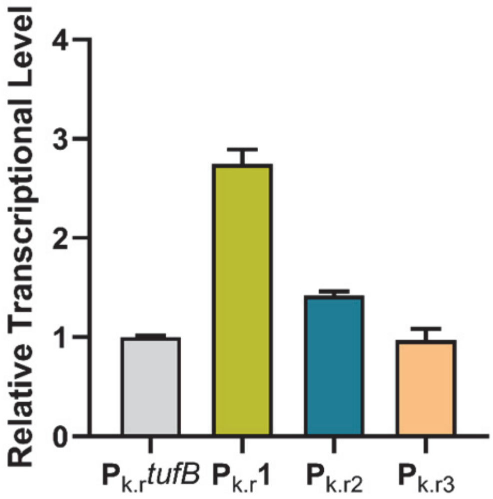

Figure S3. The relative transcriptional level of *gfp* gene initiated by different promoters in engineered *K. robustum* SPU_B003. Data represent the mean ± SD of 3 replicate.

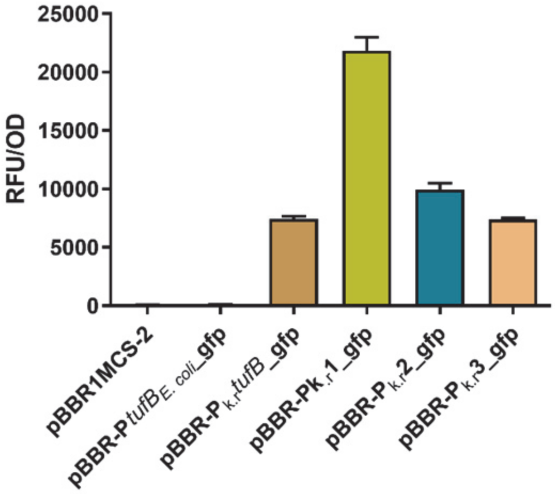

Figure S4. The whole cell relative fluorescence intensity of different recombinant *K. vulgare* SPU B805 strains. Data represent the mean ± SD of 3 replicates

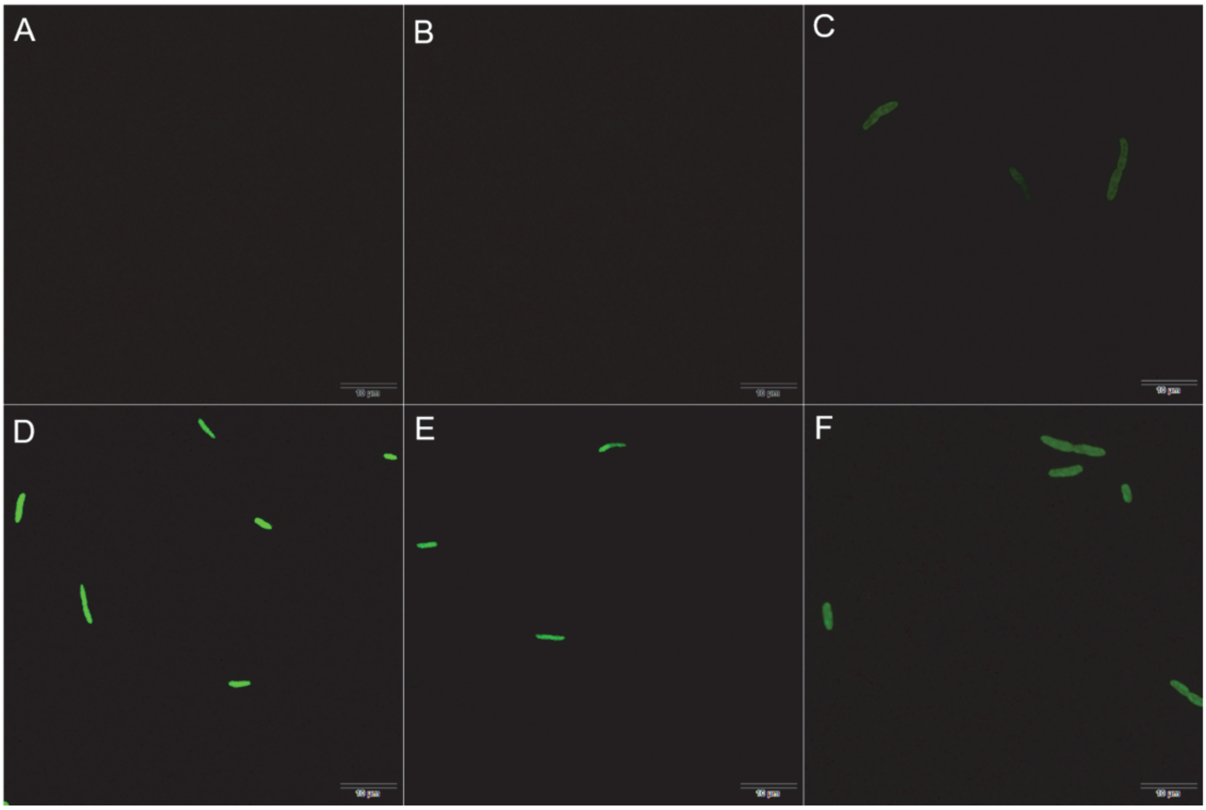

Figure S5. Fluorescence observation of recombinant *P. putida* KT2440 strains. Strains harbored plasmids of (A) pBBR1MCS-2, (B) pBBR-P$tufB_{E.\ coli}$_*gfp*, (C) pBBR-P$_{k.r}$*tufB*_*gfp*, (D) pBBR-P$_{k.r}$1_*gfp*, (E) pBBR-P$_{k.r}$2_*gfp* and (F) pBBR-P$_{k.r}$3_*gfp* were photographed with a fluorescence microscope.

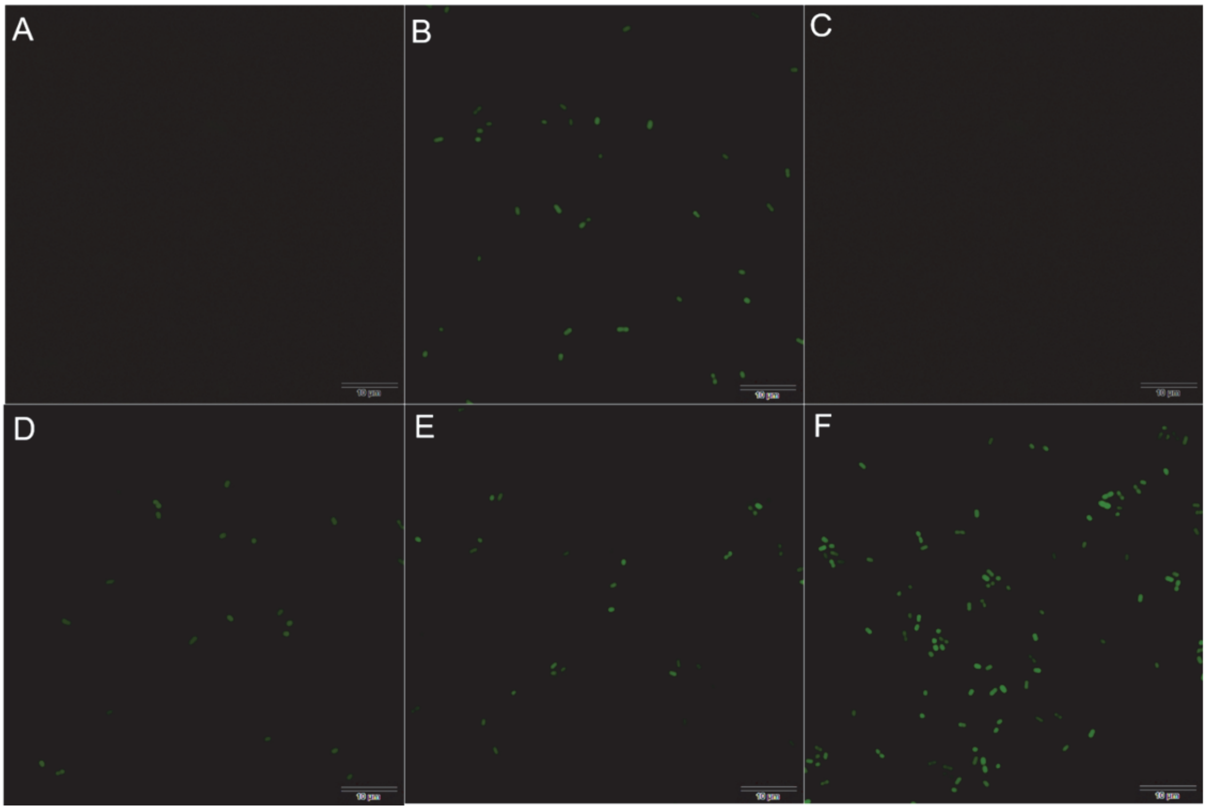

Figure S6. Fluorescence observation of recombinant *P. denitrificans* PD1222 strains.

Strains harbored plasmids of (A) pBBR1MCS-2, (B) pBBR-P*tufB*$_{E. coli}$_*gfp*, (C) pBBR-P$_{k.r}$*tufB*_*gfp*, (D) pBBR-P$_{k.r}$1_*gfp*, (E) pBBR-P$_{k.r}$2_*gfp* and (F) pBBR-P$_{k.r}$3_*gfp* were photographed with a fluorescence microscope.

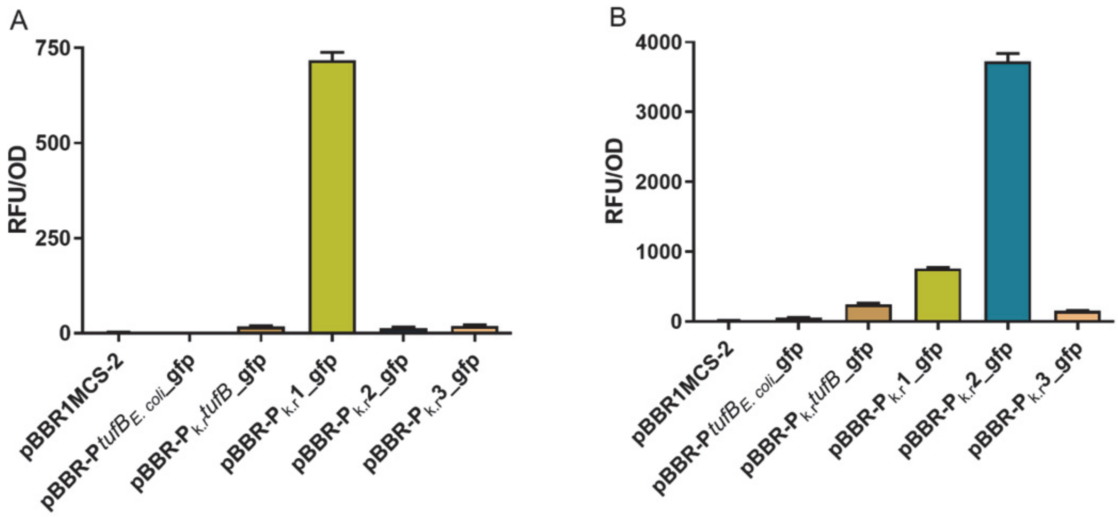

Figure S7. The whole cell relative fluorescence intensity of different recombinant strains. (A) and (B) were recombinant *B. licheniformis* and *R. ornithinolytica* harbored different recombinant plasmids. Data represent the mean ± SD of 3 replicates.

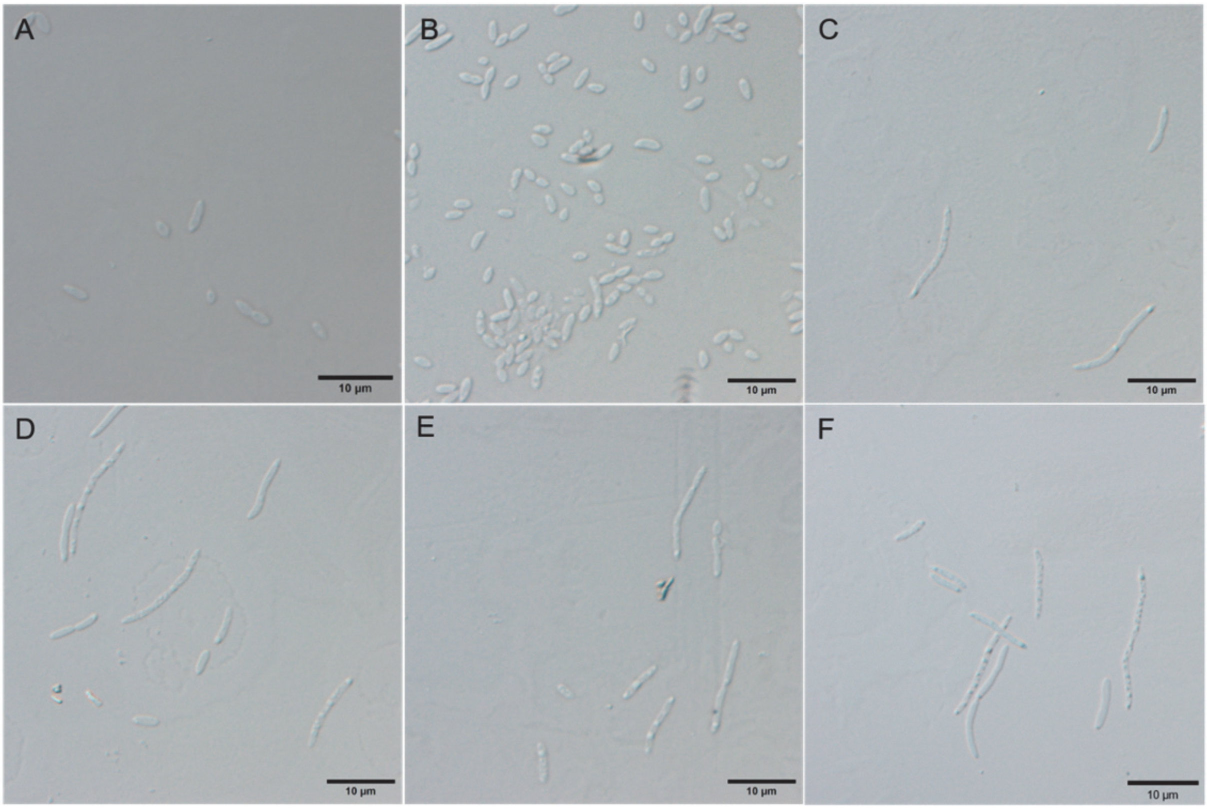

Figure S8. Morphological observation of recombinant *P. putida* KT2440 strains. Strains harbored plasmids of (A) wild-type, (B) pBBR1MCS-2, (C) pBBR-$P_{k.r}$*tufB_gfp*, (D)pBBR-$P_{k.r}$1_*gfp*, (E) pBBR-$P_{k.r}$2_*gfp* and (F) pBBR-$P_{k.r}$3_*gfp* were photographed with a DIC mode.

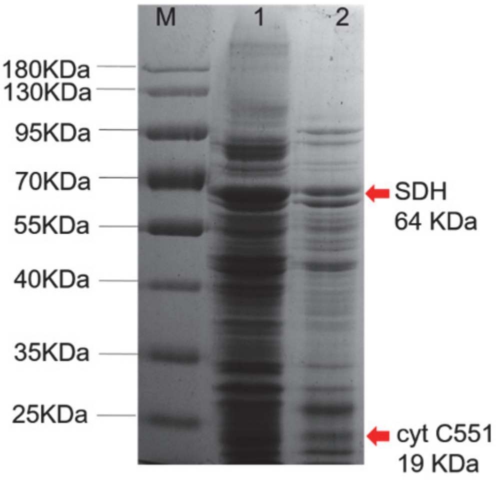

Figure S9. The expression of SDH and cyt C551 detected by SDS-PAGE. M, PageRuler Prestained Protein Ladder; 1, *K. robustum* SPU_B003 harbored plasmid pBBR-$P_{k.r}$1_*sdh*-$P_{k.r}$2_*cyt c551*; 2, *K. robustum* SPU_B003.

References

1. Wang CY, Li Y, Gao ZW, Liu LC, Zhang MY, Zhang TY,et al. 2018. Establishing an innovate carbohydrate metabolic pathway for efficient production of 2-keto-L-gulonic acid in *Ketogulonicigenium robustum* initiated by intronic promoters. *Microb. Cell Fact.* **17:**81.

2. Wang CY, Li Y, Gao ZW, Liu LC, Wu YC, Zhang MY, et al. 2018. Reconstruction and analysis of carbon metabolic pathway of *Ketogulonicigenium vulgare* SPU B805 by genome and transcriptome. *Sci. Rep.* **8:**17838.
